# Part II. Comparison of Neurodevelopmental Outcomes Between Normothermic and Hypothermic Pediatric Cardiopulmonary Bypass

**DOI:** 10.3389/fped.2019.00447

**Published:** 2019-11-05

**Authors:** Claire E. Hannon, Zachary Osman, Cathy Grant, Emma M. L. Chung, Antonio F. Corno

**Affiliations:** ^1^Department of Cardiovascular Sciences, University of Leicester, Leicester, United Kingdom; ^2^Department of Paediatric Neuropsychology, Nottingham University Hospital NHS Trust, Nottingham, United Kingdom; ^3^NIHR Leicester Cardiovascular Research Centre, Leicester, United Kingdom; ^4^Department of Medical Physics, University of Leicester, Leicester, United Kingdom; ^5^East Midlands Congenital Heart Centre, University Hospitals of Leicester NHS Trust, Leicester, United Kingdom

**Keywords:** pediatric heart surgery, hypothermia, normothermia, neurodevelopmental outcomes, cardiopulmonary bypass, congenital heart defect

## Abstract

**Objectives:** In the previous study we demonstrated that normothermic cardiopulmonary bypass (N-CPB, ≥35°C) provided better early clinical outcomes compared to mild/moderate hypothermic cardiopulmonary bypass (H-CPB, 28–34°C) for congenital heart surgery. In this follow-up study we compare early neurodevelopmental outcomes 2–3 years post-surgery.

**Methods:** In this retrospective, non-randomized observational study, the medical notes of children from our previous cohort were reviewed after 2–3 years. Demographic and neurodevelopmental outcomes were tabulated to enable blinded statistical analysis comparing outcomes between N-CPB and H-CPB surgery for congenital heart defects. Multivariate logistic regression models were developed to identify any differences in outcomes after adjustment for confounders.

**Results:** Ninety-five children who underwent H-CPB (*n* = 50) or N-CPB (*n* = 45) were included. The proportions of patients with one or more adverse neurodevelopmental outcomes 2–3 years later were 14/50 (28.0%) in the H-CPB group and 11/45 (24.4%) in N-CPB, which was not significantly different between groups (*p* = 0.47). The two CPB groups were balanced for demographic and surgical risk factors, with the exception of genetic conditions. A higher incidence of H-CPB patients acquired learning difficulties [23.1% compared to 2.56% for N-CPB (*p* = 0.014)] and neurological deficits [30.8% compared to 7.69% for N-CPB (*p* = 0.019)], but these differences were not robust to adjustment for genetic syndromes.

**Conclusions:** Our study did not reveal any significant differences in early neurodevelopmental outcomes between H-CPB or N-CPB surgery for congenital heart defects. The most important factor in predicting outcomes was, as expected, the presence of a genetic syndrome. We found no evidence that CPB temperature affects early neurodevelopmental outcomes.

## Introduction

Since the introduction of cardiopulmonary bypass (CPB) for congenital heart surgery in 1952, technology and techniques have greatly improved (1). Congenital heart surgery is a constantly evolving speciality and new techniques are often trialed and implemented rapidly with little evaluation of long-term safety or neurodevelopmental side effects. Substantial improvements in peri-operative management have led to survival rates increasing dramatically over the past few decades, especially in pediatric patients (2). Now, the focus has shifted to improving neurodevelopmental outcomes of children with congenital heart defects (CHD) which require surgical treatment (3–6). Some have termed this “Congenital Brain Disease,” finding that as children with CHD progress to school-age, they are at a higher risk of long-term neurological findings–including motor, speech and language delays, and deficits in executive functioning (3).

Some studies have hypothesized that CPB temperature may affect the neurodevelopmental outcomes of CHD patients. The theory underpinning the use of hypothermic cardiopulmonary bypass (H-CPB) is that lowering body temperature reduces the metabolic demand of the brain, providing neuroprotection (7). However, the optimum temperature for surgery (if any) is not known, therefore a single temperature has not been universally adopted and the impact of hypothermic CPB vs. normothermic surgery (conducted as close to normal body temperature) on patient's peri-operative recovery, mid-, and long-term outcomes is unknown. There are various levels of hypothermia: deep hypothermia (<28°C), without or with circulatory arrest (DHCA), with or without continuous cerebral perfusion; moderate hypothermia (28–32°C) and mild hypothermia (32–35°C). Advantages of DHCA include providing the surgeon a bloodless operating field (8) and aiming to create an isoelectric EEG, which in theory helps the patient to better tolerate cerebral hypoxia and ischemia. However, some studies suggest that cooling alone is insufficient for cerebral protection, with a study in humans and swine finding that cooling only lead to isoelectricity in the EEG of 1/10 of the human participants, at a very low temperature of 20.2°C (9).

Furthermore, the reduced flow rate and longer bypass time associated with H-CPB can lead to additional clinical sequelae. H-CPB has been found to increase the risk of cerebral edema (10, 11), disrupt cerebral autoregulation (7), and at a cellular level, disrupts enzymes, weakens cell membrane stability, and reduces ATP production, resulting in serious neurological damage at a cellular level (12). Long-term studies in children who have undergone DHCA or low-flow H-CPB, demonstrate a higher incidence of post-operative seizures, impaired motor skills at 1 year, and although a normal IQ is present, other neurodevelopmental problems were noted, even after 8 years follow up (13).

Over the last few decades N-CPB techniques have become increasingly popular. The rationale for using N-CPB is that, by keeping patients as close as possible to physiologically “normal” conditions, inflammation is minimized with a reduced need for ultrafiltration and pH regulation. This simplifies the CPB process, and as pH changes are reduced, the oxygen dissociation curve stays within normal limits, improving oxygen tissue delivery (12). These positive effects are amplified if N-CPB is conducted at a normal flow rate and without hemodilution—which is commonly utilized in H-CPB (14–16). Mounting evidence (17, 18) suggests that normothermic patients benefit from improved post-operative organ recovery, reduced blood loss (and therefore less reliance on blood products) and a quicker recovery—with reduced vasoactive inotropic score, earlier extubation, and shorter Intensive Care Unit (ICU) stays, as reported also in our original study (18). This has led to N-CPB increasing in popularity for congenital heart surgery (19–24). However, as other studies have found no significant benefits in short (12, 25–27) and long-term (12) outcomes in favor of normothermic CPB, in the absence of evidence from a Randomized Controlled Trial (RCT), and epidemiological long-term assessment of the safety and efficacy of N-CPB, the choice of CPB temperature remains controversial.

To date, no studies have directly compared neurodevelopmental outcomes between mild/moderate hypothermia and normothermic CPB. This study provides a preliminary investigation comparing normothermic surgery with mild/moderate hypothermic CPB on neurodevelopmental outcomes, ~2–3 years following surgery, to provide an initial assessment as to whether N-CPB has a significantly different safety profile in comparison to H-CPB. Demographic and clinical data were collected to retrospectively review whether any specific patient or operative factors were associated with a higher risk of early neurodevelopmental delay in children under 5.

## Methods

### Data Collection

This retrospective cohort study aimed to compare early (3–5 years) neurodevelopmental outcomes, as diagnosed by psychologists and neurodevelopmental specialists in children who had undergone H-CPB or N-CPB congenital heart surgery as infants (<2 years at the time of surgery). Hospital medical records were reviewed for patients from our previous cohort who underwent either N-CPB or H-CPB surgery, conducted during a 2 years transition to N-CPB in our center between January 2014 and December 2015. All data from the previous study, including the demographic characteristics of the patients, diagnosis, risk stratification, type of surgical procedure, and all the immediate clinical outcomes have already been reported (18).

### Selection Criteria

This study follows the same patient population as described in our previously published study on clinical outcomes of CHD patients immediately post-operatively, where all details relative to the patients diagnosis, type of surgery, duration of cardiopulmonary bypass and aortic cross clamp, and post-operative outcomes were reported in details (18). In that study, infants were excluded from evaluation if they met one or more of the following exclusion criteria:

- >2 years of age at the time of surgery
- premature <38 weeks of gestational age
- surgical procedure not requiring CPB
- CPB with deep hypothermia (temperature <28°C)
- any period of circulatory arrest during the operation
- aortic arch hypoplasia/interruption requiring aortic arch reconstruction

The data collector for this study on neurodevelopmental outcomes in the same cohort of patients of the previous clinical study was blinded to CPB temperature, which was added following statistical analysis. Follow-up clinic notes were reviewed for information on any recorded early neurodevelopmental outcomes 2 years after the patient's first surgery. The data collector recorded the outcomes from the most recent follow-up appointment at the time of data collection, to give enough time for any early neurodevelopmental delays to have become evident.

The following information was gathered: clinical information at the time of surgery (age, sex, weight, prematurity, presence of genetic syndromes, and pre-operative cyanosis). A measure of pre-operative surgical risk and mortality was calculated for each group in the form of a STAT risk stratification score (on a scale from 1–5) (28). The CPB type (N-CPB or H-CPB) was recorded as Group A or B to facilitate blinded statistical analysis.

Potential confounders included genetic conditions known to impact neurodevelopment. In our cohort, diagnoses included Down syndrome, DiGeorge syndrome and various 22q deletion syndromes. Prematurity, low weight, and the degree of pre-operative cyanosis were recorded, as these factors could be associated with growth restriction, cerebral hypoxia and impaired brain development prior to surgery. The presence of post-operative cyanosis (such as in children who underwent central shunt or bidirectional Glenn anastomosis on CPB) and age at the time of assessment were also noted, as these factors may influence the risk of either acquiring or reporting neurodevelopmental outcomes.

The neurodevelopmental outcomes were recorded after the diagnosis was performed with consistent analysis by psychologists and neurodevelopmental specialists, and included neurodevelopmental, motor, language or growth delay, neurological deficits, and learning or behavioral difficulties. The neuromotor deficits were considered in the evaluation of motor delays, neurological deficits and neurodevelopmental delays. The notes were also searched for evidence of any support required for neurodevelopmental problems, such as physiotherapy, speech therapy, additional help in school, special educational needs support (SEN), or having an education, health and care plan (EHCP) in place. Observations relating to school-age children were not included in our analysis, as the focus of this study was on early (pre-school) neurodevelopmental outcomes, and the majority of children had not yet reached school age.

This service safety evaluation study was reviewed and approved by the University Hospitals of Leicester NHS Trust.

On review of the medical records, children with missing data or clinical notes (e.g., due to leaving the care of the hospital) were excluded from further analysis. Those who were over 2 years of age at the time of surgery were also excluded as we wished to focus on the impact of surgery in infants, who are thought to be more at risk of neurological complications. Premature neonates were excluded but ex-premature infants at the time of surgery were eligible. Patients with a genetic diagnosis, already included in the previous cohort for the evaluation of early clinical outcomes (18), were initially included in our analysis, but were then adjusted for using a multivariate logistic regression model, as clearly detailed in the following statistical plan.

**Figure 1:**
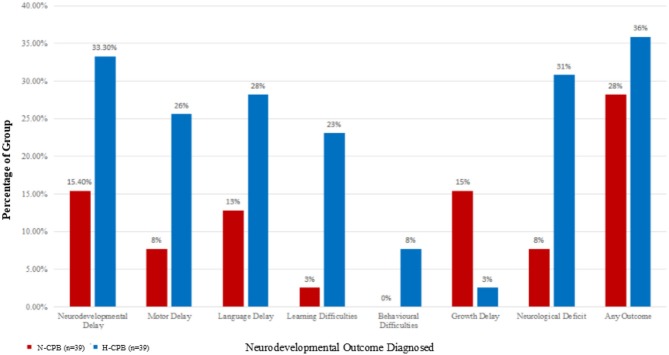
The percentages of each group who have been diagnosed with an adverse neurodevelopmental outcome a few years after surgery.

The surveillance cohort identified from our previously published study included 99 patients. Of these, four were excluded due to a lack of follow-up clinic notes due to relocation. Of the remaining 95 patients (45 N-CPB and 50 H-CPB), a further 17 were excluded from the current analysis due to incomplete or unavailable data on evaluation by neurodevelopmental specialists. Data from these 17 patients were included only in the initial demographic comparison for completeness of the study but were not included in the analysis of the neurodevelopmental outcomes. Table 1 compares the initial cohort demographics and the incidence of potential confounders between the two groups. Table 2 summarizes details for the sub-cohort used for analysis of outcomes, confirming that homogeneity was maintained once the patients with missing data were excluded. Table 3 gives an overview of the incidence of adverse neurodevelopmental outcomes for each group and an infographic summarizing differences between the N-CPB and H-CPB groups is provided as Figure 1.

**Table 1:** Patient demographics.

	**Total cohort**		
**Parameter**	**N-CPB (*n* = 45)**	**H-CPB (*n* = 50)**	***p*-value**
Age (Days)	Median: 205 (143–310)	Median: 187.5 (73–284)	0.67^*^
Weight (kg)	Median: 6.1 (4.2–7.6)	Median: 6.15 (4.1–8)	0.97^*^
Born prematurely (*n* = 39)	6 (15.4%)	3 (7.69%)	0.31^F^
Sex			0.81^T^
• Male	25 (55.6%)	29 (58%)	
• Female	20 (44.4%)	21 (42%)	
Pre-operative cyanosis			0.83^F^
• None	24 (53.3%)	25 (50%)	
• Mild	10 (22.2%)	13 (26%)	
• Moderate	10 (22.2%)	9 (18%)	
• Severe	1 (2.22%)	3 (6%)	
Genetic conditions			0.009^F^
Post-operative cyanosis	1 (2.22%)	10 (20%)	0.339^F^
• None	40 (88.9%)	48 (96%)	
• Mild	1 (2.22%)	1 (2%)	
• Moderate	4 (8.89%)	1 (2%)	
• Severe	0	0	
Presence of a comorbidity?	12 (31%)	19 (49%)	0.105^T^
Multiple surgeries	7 (16%)	12 (24%)	0.502^F^

* *Mann Whitney U-test*.

T *Chi^2^ test*.

F *Fisher's exact test*.

**Table 2:** Patient demographics of those included in the analysis.

	**Analysis cohort**		
**Parameter**	**N-CPB (*n* = 39)**	**H-CPB (*n* = 39)**	***p*-value**
Age (Days)	Median: 205 (35–310)	Median: 197 (74–284)	0.908^*^
Weight (kg)	Median: 6.1 (3.9–7.6)	Median: 6.3 (4.1–8)	0.497^*^
Born prematurely	6 (15.4%)	3 (7.69%)	0.481^T^
Sex			0.361^T^
• Male	20 (51%)	24 (62%)	
• Female	19 (49%)	15 (38%)	
Pre-operative cyanosis			0.857^F^
• None	18 (46.2%)	17 (43.6%)	
• Mild	10 (25.6%)	10 (25.6%)	
• Moderate	10 (25.6%)	9 (23.1%)	
• Severe	1 (2.6%)	3 (7.7%)	
Genetic conditions			0.007^F^
Post-operative cyanosis	1 (2.6%)	10 (25.6%)	0.512^F^
• None	34 (87.2%)	39 (94.9%)	
• Mild	1 (2.6%)	1 (2.6%)	
• Moderate	4 (10.3%)	1 (2.6%)	
• Severe	0	0	
Presence of a comorbidity?	12 (31%)	19 (49%)	0.105^T^
Multiple surgeries	7 (18%)	12 (31%)	0.454^F^

* *Mann Whitney U-test*.

T *Chi^2^ test*.

F *Fisher's exact test*.

**Table 3:** Neurodevelopmental outcomes of the analysis cohort.

	**N-CPB (*n* = 39)**	**H-CPB (*n* = 39)**	***p*-value**
Neurodevelopmental delay	6 (15.4%)	13 (33.3%)	0.065^T^
Language delay	5 (12.8%)	11 (28.2%)	0.092^T^
Motor delay	3 (7.69%)	10 (25.6%)	0.065^F^
Learning difficulties^*^	1 (2.56%)	9 (23.1%)	0.014^F^
Behavioral difficulties	0	3 (7.69%)	0.24^F^
Neurological deficit^*^	3 (7.69%)	12 (30.8%)	0.019^F^
Growth delay	6 (15.4%)	1 (2.56%)	0.108^F^

* *Mann Whitney U-test*.

T *Chi^2^ test*.

F *Fisher's exact test*.

### Statistical Analysis

Statistical analysis was performed using Stata 15 (StataCorp, TX, USA), with the analyst blinded to whether patients had undergone N-CPB or H-CPB; labeled as “Group A” or “Group B.” For clarity, the groups have been unblinded for presentation of the findings.

Firstly, tests for normality and exploratory summary statistics were generated for each group and traditional hypothesis tests used to identify any heterogeneity in characteristics between groups. These included a Mann-Whitney test for continuous non-parametric variables, and a Chi^2^ test (or Fisher's exact test, as appropriate) for categorical data, based on a null hypothesis of no difference between N-CPB and H-CPB groups. Confounders that were not homogeneously distributed between the two groups, with *p* < 0.1 as the level of significance (e.g., genetic conditions) were noted as requiring further investigation using multivariate regression analyses. Age and weight at the time of surgery were also investigated as confounders for our model, as previous studies have demonstrated a link between these factors and outcomes. Other potential confounding factors (including cyanosis parameters, multiple surgery, sex, age at follow-up, STAT score, and premature birth) were either homogeneously distributed between groups, or heavily underpowered, so were not incorporated into further models.

Odds Ratios (ORs) and 95% confidence intervals (CIs) were estimated using univariate logistic regression for each outcome variable (developmental delay, motor delay, language delay, growth delay, neurological deficit, learning difficulties, and behavioral difficulties) with the factor of interest (N-CPB vs. H-CPB) as the explanatory variable. This univariate analysis was used to provide an initial assessment of whether there were any statistical differences in outcomes when considering H-CPB and N-CPB in the absence of adjustment for confounders.

Multivariate logistic regression was then performed to assess whether the findings were robust to adjustment for confounding genetic conditions known to affect developmental outcomes, age and weight. Revised Odds Ratios (ORs) and 95% CIs relating to the impact of N-CPB or H-CPB after adjustment for known confounders are presented.

## Results

Overall, infants in the final surveillance cohort were a median of 6 months, 1 week old (IQR: 2 months 1 week, 9 months 2 weeks) at the time of surgery with a median weight at the time of surgery of 6.1 kilograms (IQR 4.1–7.7). Forty-one patients were female (43%), 54 (57%) were male, and 11 (12%) had genetic conditions known to affect brain development. The median time from surgery to the patient's most recent follow-up visit was 2 years and 9 months (IQR: 2 years 2 months−3 years 5 months), corresponding to a mean age at the time of assessment of 3 years, 3 months (SD ±:1 year, 2 weeks). By this time, 25/78 children (32%) whose outcomes had been recorded developed a neurological or developmental problem, 11 (44%) of patients were from the N-CPB group and 14 (56%) from the H-CPB group, which was not statistically different between groups (*p* = 0.47). In total, 20 of the 78 children (26%) experienced multiple adverse neurodevelopmental outcomes (N-CPB: 7 children, H-CPB: 13 children).

Following our original statistical plan, Mann-Whitney and Chi^2^ tests (or Fisher's exact test where appropriate) were performed. These showed no significant differences in age or weight at the time of surgery, sex, prematurity, and the presence of cyanosis, comorbidities or multiple surgeries between groups. A statistically significant difference between groups was observed for the presence of a genetic condition, which was higher in the H-CPB group; affecting 10/39 (25%) of H-CPB infants compared to only 1/39 (3%) in the N-CPB group. The STAT score from our previous study demonstrated no significant difference in the risk of surgery between the two groups. The STAT score for N-CPB was 2 ± 1.2 compared to 2 ± 1.1 for H-CPB, *p* = 0.46 (18).

Univariate logistic regression analysis was used to compare outcomes between groups (N-CPB vs. H-CPB) in the absence of adjustment for confounders, see Table 4. This suggested significant differences (at *p* < 0.05) between the two groups for motor delay, learning difficulties and diagnosed neurological deficits.

**Table 4:** Univariate exact logistic regression models: the effect of group on the neurodevelopmental outcomes.

**CPB group**	**Odds ratio**	**95% confidence interval**	***p*-value**
Model 1: NDD	2.63	0.90, 7.62	0.076
Model 2: motor delay	3.71	1.01, 13.7	0.049^*^
Model 3: language delay	2.53	0.82, 7.84	0.107
Model 4: learning difficulty	7.99	1.34, 47.7	0.023^*^
Model 5: behavioral difficulty	7.57	0.38, 151.7	0.185
Model 6: growth delay	0.20	0.032, 1.26	0.081
Model 7: neurological deficit	4.74	1.31, 17.13	0.018^*^

*NDD, Neurodevelopmental delay*.

* Statistically significant (P < 0.05)

### Multivariate Analysis

A multivariate logistic regression model was then implemented to allow adjustment for confounders. As genetic condition perfectly predicted outcome for some models (e.g., learning difficulties), and considering our small sample size, an exact logistic regression model (using a user-generated function: firthlogit) was implement to obtain exact Odds Ratios and 95% CIs. Following adjustment for genetic conditions, the difference between the N-CPB and H-CPB groups became non-significant for all neurodevelopmental outcomes. The final multivariate model *R*^2^ values, estimated Odds Ratios and 95% CI, *p*-values developed to describe each of the outcomes of interest are summarized in Table 5.

**Table 5:** Multivariate exact logistic regression models: the effect of group allocation on neurodevelopmental outcomes.

**CPB group**	**Odds ratio** **(95% CI)**	***p*-value**	**Adjusted** **R**^**2**^
Model 1: neurodevelopmental delay	0.752 (0.18, 3.22)	0.701	0.0254
Model 2: motor delay	0.60 (0.08, 4.63)	0.624	0.0006
Model 3: language delay	0.438 (0.06, 2.96)	0.397	0.0114
Model 4: learning difficulties	1.57 (0.04, 61.6)	0.809	0.0015
Model 5: behavioral difficulties	7.378 (0.35, 154.4)	0.198	0.630
Model 6: growth delay	0.305 (0.05, 1.96)	0.211	0.306
Model 7: neurological deficit diagnosed	1.245 (0.2, 7.86)	0.816	0.0069

**Figure 2:**
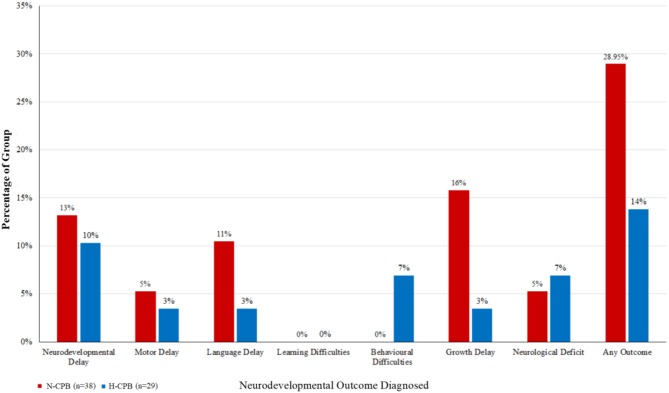
The percentages of each group diagnosed with an adverse neurodevelopmental outcome a few years after surgery, excluding the cohort of patients who have been diagnosed with a genetic syndrome.

As a sensitivity analysis, subjects with genetic conditions were also excluded from our analysis from the outset, and the analysis repeated. Homogeneity between groups was maintained, however on univariate analysis, differences between groups were not statistically significant between N-CPB and H-CPB (Figure 2). This provided further confirmation that CPB group had no statistically significant effect on neurodevelopmental outcomes in the absence of genetic factors.

Age and weight (at the time of surgery) were included in our model, as previous clinical research suggests that these factors could affect outcome. We found that neither age or weight were statistically significant for any of the outcomes we tested at a significance level of *p* < 0.05. Age and weight were of borderline significance for the development of growth delay; age: *p* = 0.073, Weight: *p* = 0.103. However, logistic regression suggests an Odds Ratio for the effect of age very close to 1, which suggests the effect, if present, is small (i.e., that for each additional day the child was older, the risk of developing growth delay increases by 0.8% (OR: 1.008, 95% CI: 1.00, 1.02) which corresponds to an increased risk of 5.6% for each week older (OR 1.056, 95% CI 1.00, 1.12). For each additional kilogram the risk of delay almost halves (OR: 0.54, 95% CI 0.26, 1.13), although the confidence limits on this estimate are wide and include 1, so there could be no difference. These trends suggest that older infants with lower body weight at the time of surgery may be more likely to experience growth delay.

## Discussion

This study directly compares neurodevelopmental outcomes, as diagnosed by psychologists and neurodevelopmental specialists, 2–3 years after surgery in infants who underwent CHS using either normothermic or mild/moderate hypothermic CPB, following the previous study where only the immediate clinical outcomes were compared (18). Information on neurocognitive outcomes were obtained for 78 children enrolled in a previous study examining immediate clinical outcomes post-surgery. After adjustment for genetic factors, no significant difference was detected in neurodevelopmental outcomes between N-CPB and H-CPB patients. Our findings provide reassurance that the introduction of N-CPB at our center did not significantly worsen early neurodevelopmental outcomes. With previous studies demonstrating that immediate post-operative recovery is substantially improved by the use of N-CPB (17–24), this provides additional evidence in support of N-CPB adoption.

Like many studies in this area, this small single-center neurodevelopmental safety evaluation is associated with a number of methodological limitations:

- Although the N-CPB and H-CPB groups were of roughly equal size, and broadly homogeneous for surgical and demographic risk factors, blinded randomization was not used to allocate patients to H-CPB and N-CPB. We are therefore unable to exclude selection or recall bias from our study. A strength of our study is that both our researcher and data collector were blinded to the parameter of interest (N-CPB or H-CPB) which reduced the risk of bias in our analysis.
- Our study was confined to a single-center and complete sets of outcome data were only obtained for 78 patients, resulting in a sample size that was too small to reach reliable conclusions; a non-significant difference between groups may have been due to lack of power rather than true lack of difference. The power of our study was estimated to be 11%. This pilot data remains valuable for power calculation purposes; a sample size of around 575 in each group would be required to achieve a power of 80%. Future larger comparisons of N-CPB vs. H-CPB would be needed to confidently determine whether there is a difference in outcomes between the two groups.
- Our study was observational and did not involve follow-up of patients through formal neuropsychological evaluation. Data were extracted from medical notes written by the consultant cardiologist at the time of surgery and at follow-up. Cardiologists may not have explicitly looked for neurodevelopmental problems or used any formal testing. No standard screening is implemented and clinician's interpretation of difficulties and delays can vary. Neurodevelopmental problems are normally followed-up by community pediatricians, and may therefore not have been noted in cardiology hospital records. This approach has potential to lead to under-reporting of neurological problems in CHD patients.
- Our follow up occurred 2–3 years after surgery, which may be a too short an interval time for neurodevelopmental delays to emerge. Future research should include formal evaluation of outcomes at 3–5 years and 11–12 years.

Our findings illustrate the importance of adjusting for patient-specific factors, such as genetic syndromes, when analyzing differences in neurodevelopment outcomes between groups. Since our H-CPB group included a higher proportion of patients with genetic conditions, a univariate comparison suggested an adverse effect of H-CPB on neurodevelopmental outcomes, which would have been misleading, and was not robust to adjustment for genetics. The main diagnoses we encountered were Down and DiGeorge syndrome, with the other patients being diagnosed with various deletion syndromes. These results confirm previous research where the presence of a genetic abnormality was associated with significantly poorer neurodevelopmental outcomes at 1 year of age (29). A statement in 2012 by the American Heart Association (AHA) suggested identifying high-risk patients for follow-up. High risk is classed as any neonate or infant requiring open heart surgery, children with cyanotic lesions, or those who had CHD with a co-morbidity or genetic syndrome (30). These types of patients have the highest prevalence of neurodevelopmental defects and therefore require close follow-up. The AHA recommends that all high-risk patients be referred for formal developmental evaluation and also re-evaluated for developmental delays at 12–24 months, 3–5 years, and 11–12 years. This would ensure that neurodevelopmental delays are recognized and supported earlier. For lower risk infants standard screening tools would be utilized before referral is required (29).

Care should be taken to identify and adjust for relevant patient factors affecting the likelihood of neurodevelopmental delay. Although no explanatory variables, other than genetics, were found to have a statistically significant effect on the neurodevelopmental outcomes in our cohort, other patient and societal factors known to impact development, should also be considered in future research. Other than genetic factors, our groups were homogeneous for other clinical details, and it is hoped that any unidentified confounders would have been homogeneously distributed between groups.

A borderline association between growth delay and infants with low weight for their age at the time of surgery was observed in our data. This is not unexpected and agrees with previous studies (29) suggesting a greater delay between birth and surgery may be associated with a decrease in brain tissue oxygenation due to the heart defect, which increases the risk of white matter injury (31). These brain injuries have potential to contribute to neurodevelopmental delays, such as motor deficits and behavioral disorders (32). Other studies have suggested that younger patients are more vulnerable to brain injury (4, 33) due to the immature brain being unable to cope with changes induced in the cerebral circulation associated with bypass surgery. Therefore, delaying surgery in order to allow the brain time to mature must be weighed against the risk of prolonged exposure to pre-operative hypoxia in this subset of patients.

As we limited our comparison between N-CPB, ≥35°C and mild/moderate H-CPB, 28–34°C, the conclusions of this study do not apply to the use of DHCA. Current evidence suggests that lower degrees of hypothermia, with or without flow reduction or circulatory arrest, are associated with a significantly increased risk of neurological complications (7–16).

## Limits of the Study

The major limits of this study have already been indicated above: retrospective, single center, and based on a small number of a previous cohort of patients, and 11/78 (14.1%) patients with genetic syndromes known to affect brain development: Down syndrome, DiGeorge syndrome, and q22 deletion.

We didn't use any device, such as Trans-Cranial Doppler (TCD) to detect the presence of microbubbles, potentially causing damage to the cerebral microcirculation, with consequences on the neurodevelopmental outcomes. The TCD has not yet reported as introduced in the daily clinical practice in pediatric cardiac surgery, and certainly it was not available in our unit at the time of this study. Nevertheless, we have recently performed a pilot study with TCD in children under 2 years of age undergoing CPB, with interesting preliminary observations, and we planned to introduce it the clinical practice as additional tool for intra-operative neuromonitoring with a prospective data collection.

## Conclusions

This study did not detect any significantly increased risk of developing neurological complications following N-CPB surgery. One of the most important factors in predicting adverse neurological and developmental outcomes was the presence of a genetic condition. Following adjustment for genetic conditions, the incidence of neurodevelopmental complications was broadly similar between the groups. Trends observed with age and weight add to evidence that the time until surgery could be an important factor to consider for patients undergoing CPB surgery. Future studies should have a longer follow-up time, involving formal testing by a neuropsychologist, and ideally take the form of a prospective multi-center randomized control trial between N-CPB and H-CPB.

## Data Availability Statement

The raw data supporting the conclusions of this manuscript will be made available by the authors, without undue reservation, to any qualified researcher.

## Author Contributions

CH contributed to the design of the study, the preparation of the database, the data collection and analysis, and the preparation of the manuscript. ZO contributed to the preparation of the database and the data collection and analysis and revised the manuscript. CG and EC contributed to the design of the study, the preparation of the database, the data analysis, and the preparation of the manuscript. AC contributed to the design of the study, the preparation of the database, the data analysis, and the revision of the manuscript.

### Conflict of Interest

The authors declare that the research was conducted in the absence of any commercial or financial relationships that could be construed as a potential conflict of interest.
